# Atrial fibrillation burden and oral anticoagulation: a context-dependent framework for net clinical benefit beyond binary AF definitions

**DOI:** 10.3389/fcvm.2026.1859191

**Published:** 2026-07-02

**Authors:** Zonghong Wu, Jie Hao

**Affiliations:** Department of Cardiology, The Second Hospital of Hebei Medical University, Shijiazhuang, China

**Keywords:** atrial fibrillation burden, bleeding risk, device-detected atrial fibrillation, net clinical benefit, oral anticoagulation, thromboembolic risk

## Abstract

Atrial fibrillation (AF) is a major risk factor for ischaemic stroke, and oral anticoagulation (OAC) effectively reduces thromboembolic risk in patients with clinically diagnosed AF. With the expanding use of continuous rhythm monitoring and wearable devices, increasing numbers of device-detected atrial arrhythmias are being identified, challenging the traditional binary approach to AF diagnosis and anticoagulation decision-making. AF burden, commonly defined as the proportion of monitored time spent in AF during a defined monitoring period, has emerged as a quantitative measure of AF exposure. Observational studies using continuous monitoring, including ASSERT, TRENDS, and KP-RHYTHM, consistently demonstrate that increasing AF burden is associated with progressively higher absolute risks of ischaemic stroke and systemic embolism. However, patients with low AF burden generally have relatively low absolute event rates. Randomized trials such as LOOP, ARTESiA, and NOAH-AFNET 6 further evaluated anticoagulation in patients with device-detected or subclinical AF. Collectively, these studies suggest that, in low-burden AF populations, reductions in thromboembolic events with OAC may be offset by increased bleeding risk, resulting in limited overall net clinical benefit. Current evidence indicates that AF burden alone is unlikely to provide sufficient guidance for anticoagulation decisions. Instead, the net clinical benefit of OAC appears to depend on the interaction among AF burden, thromboembolic risk, bleeding risk, and patient-specific clinical factors. This review summarizes current evidence on AF burden and discusses how AF burden, thromboembolic risk, and bleeding risk may jointly influence the net clinical benefit of anticoagulation.

## Graphical Abstract

**Figure:**
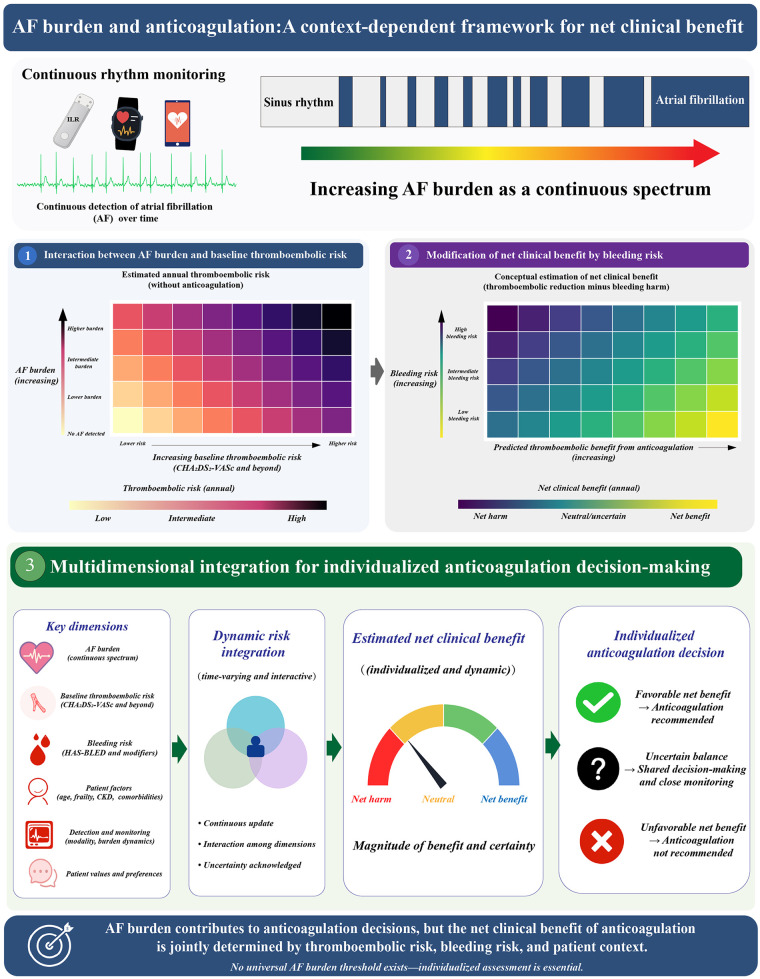

## 1 Introduction

### 1.1 Background

Atrial fibrillation (AF) is one of the most common sustained cardiac arrhythmias in clinical practice (2), and its prevalence increases markedly with age. Data from the Framingham Heart Study and other large population-based cohorts have shown that AF is associated with an approximately four- to five-fold increase in the risk of ischemic stroke compared with individuals without AF (27, 28). Randomized controlled trials and meta-analyses have consistently demonstrated that oral anticoagulation (OAC) reduces the risk of ischemic stroke in patients with AF by approximately 60%–70%, although at the cost of increased bleeding risk (34).

### 1.2 Limitations of traditional anticoagulation decision-making

In current clinical practice, decisions regarding anticoagulation are primarily guided by stroke risk stratification using the CHA_2_DS_2_-VASc score, with the presence of AF generally considered a prerequisite for treatment initiation (19). This approach implicitly assumes that stroke risk is relatively homogeneous once AF is present within a given clinical risk category (3). However, this assumption does not adequately reflect the substantial variability in AF frequency, duration, and temporal patterns among individuals (32).

### 1.3 New challenges introduced by continuous rhythm monitoring

Traditionally, AF has been treated as a categorical diagnosis based on electrocardiographic documentation of arrhythmia episodes meeting predefined criteria (2). With the increasing use of prolonged rhythm monitoring, atrial arrhythmias are now recognized as existing along a temporal continuum, ranging from brief irregular atrial activity to sustained clinical AF.

The widespread adoption of implantable pacemakers, implantable cardioverter-defibrillators, insertable cardiac monitors, and wearable monitoring devices has markedly increased the detection of previously unrecognized asymptomatic or device-detected atrial arrhythmias. In the ASSERT study, approximately 10% of patients without prior clinical AF developed device-detected atrial high-rate episodes (AHREs) within 3 months after device implantation, with most episodes being short in duration (9). The TRENDS study further demonstrated substantial temporal variability in both AF episode frequency and duration within individual patients (11).

Recent wearable-based studies have also expanded AF detection beyond traditional implantable monitoring systems. Large-scale smartwatch and wearable device studies have shown that wearable monitoring can identify previously undiagnosed AF in large community populations (7, 8). As wearable technologies continue to become more widely used, an increasing proportion of newly detected AF is likely to originate from consumer-facing or ambulatory monitoring platforms rather than conventional clinical encounters. This shift introduces additional challenges regarding the interpretation of low-burden or short-duration AF detected outside traditional clinical settings.

Prospective ambulatory ECG studies further suggest that among patients with AF-like atrial arrhythmias lasting <30 s during initial monitoring, nearly half subsequently develop AF episodes lasting ≥30 s during extended follow-up (5). Progression is more commonly observed in individuals with established thromboembolic risk factors, including elevated CHA_2_DS_2_-VASc scores, vascular disease, or prior stroke (5). These findings support the value of prolonged rhythm monitoring in selected high-risk populations and highlight the dynamic nature of atrial arrhythmias over time. Collectively, these observations underscore the limitations of short-term or single time-point rhythm assessment and emphasize the importance of continuous longitudinal monitoring for characterizing AF exposure. In this context, AF burden has emerged as a quantitative framework that extends beyond the traditional binary classification of “AF present” vs. “AF absent.”

### 1.4 Emergence of the concept of AF burden and its association with risk

According to the recent ESC Council on Stroke/EHRA consensus statement, AF burden is preferably defined as the proportion of monitored time spent in AF during a defined monitoring period and should ideally be reported together with the longest uninterrupted AF episode when clinically relevant (30).

Several observational studies have demonstrated important associations between AF burden and thromboembolic risk. The TRENDS study was among the first to introduce the concept of “daily AF burden” and reported increased thromboembolic risk among patients with higher cumulative AF exposure (11). Subsequent analyses from ASSERT showed that longer AHRE duration was associated with progressively higher risks of ischemic stroke or systemic embolism (10). Using long-term continuous monitoring data, the KP-RHYTHM study further demonstrated that AF burden remained independently associated with ischemic stroke risk after adjustment for the CHA_2_DS_2_-VASc score (12).

These findings suggest that AF burden may provide clinically relevant information beyond a simple binary AF diagnosis and may complement conventional thromboembolic risk assessment frameworks.

### 1.5 From risk association to treatment decision: A central controversy

Although observational studies consistently demonstrate associations between AF burden and stroke risk, risk association does not necessarily translate directly into treatment benefit. In patients with subclinical atrial fibrillation (SCAF) or low-burden device-detected AF, the absolute risk of ischemic stroke is generally lower than that observed in patients with clinically diagnosed AF, whereas bleeding risk associated with anticoagulation may remain clinically important regardless of AF burden level. This imbalance between potential benefit and harm creates uncertainty regarding the overall net clinical benefit of anticoagulation in these populations.

The LOOP trial evaluated a screening strategy using insertable cardiac monitors followed by anticoagulation initiation after AF detection, but did not demonstrate a significant reduction in stroke or systemic embolism (15). The study population was characterized predominantly by short-duration and low-burden AF episodes. Subsequently, the ARTESiA (17) and NOAH-AFNET 6 (16) randomized trials evaluated direct oral anticoagulants in patients with device-detected SCAF or AHREs. Collectively, these studies showed that anticoagulation may reduce ischemic stroke or systemic embolism, but at the expense of increased major bleeding, resulting in a relatively modest net clinical benefit at the population level.

Secondary analyses from ARTESiA further demonstrated that baseline AF burden, including episode frequency and longest episode duration, was not significantly associated with stroke or systemic embolism risk and did not significantly modify the relative efficacy of apixaban compared with aspirin (37). Similarly, supplementary analyses from NOAH-AFNET 6 showed no significant interaction between baseline AHRE burden and anticoagulation treatment effect. Together, these findings suggest that currently available randomized evidence does not support the use of AF burden alone to determine anticoagulation benefit.

In the present review, net clinical benefit refers to the overall balance between reduction in thromboembolic events and the increased risk of bleeding-related harm associated with anticoagulation within a specific clinical context. Importantly, this balance should be interpreted as dynamic and individualized rather than fixed or uniform across all patient populations. Stroke prevention benefit may vary according to AF burden, CHA_2_DS_2_-VASc score, age, comorbidities, and other clinical factors, while bleeding liability may also differ substantially according to baseline bleeding risk, frailty, renal dysfunction, and concomitant therapies. Accordingly, AF burden should be interpreted as one component of an integrated and context-dependent risk assessment framework rather than as an isolated determinant of anticoagulation decision-making. The central unresolved question is therefore not whether a universal AF burden threshold exists, but whether AF burden may help refine individualized estimation of net clinical benefit when interpreted together with conventional thromboembolic and bleeding risk assessment.

This review integrates evidence from continuous rhythm monitoring studies, wearable monitoring studies, observational cohorts, and randomized clinical trials to summarize the definition and measurement of AF burden, examine its associations with ischemic stroke and other clinically relevant outcomes, and discuss the evolving concept of burden-informed and individualized anticoagulation strategies. These insights may help inform future research aimed at developing more precise and patient-centered approaches to stroke prevention in AF.

Given the substantial overlap and heterogeneity among terms such as AF burden, AHRE, subclinical atrial fibrillation (SCAF), and device-detected AF in the existing literature, terminology throughout this review is primarily aligned with the 2025 ESC Council on Stroke/EHRA consensus statement and the American Heart Association scientific statement on subclinical and device-detected AF (1, 30). Standardized terminology is summarized in Supplementary Table S1.

## 2 Methods

This study was designed as a narrative review focusing on atrial fibrillation (AF) burden, subclinical atrial fibrillation (SCAF), atrial high-rate episodes (AHREs), thromboembolic risk, and anticoagulation strategies.

A structured literature search was performed using PubMed/MEDLINE and Web of Science as the primary databases. Additional relevant publications were identified through manual reference screening. Studies published up to April 2026 were considered.

The search strategy included combinations of the following terms: “atrial fibrillation burden,” “subclinical atrial fibrillation,” “atrial high-rate episodes,” “AHRE,” “device-detected atrial fibrillation,” “stroke risk,” “oral anticoagulation,” “net clinical benefit,” “cognitive decline,” “dementia,” “quality of life,” “wearable devices,” “catheter ablation,” and “continuous rhythm monitoring.”

Priority was given to randomized controlled trials, prospective cohort studies, major observational registries, international clinical guidelines, consensus statements, and clinically relevant review articles. Landmark studies, including ASSERT, TRENDS, KP-RHYTHM, LOOP, ARTESiA, and NOAH-AFNET 6, were specifically reviewed because of their major contributions to the current understanding of AF burden, device-detected AF, and thromboembolic risk.

Because research on AF burden and device-detected atrial arrhythmias is evolving rapidly, particular attention was given to studies published within the past 5 years. Earlier landmark studies were also included when considered foundational to the field.

Studies were selected according to their relevance to the following topics:

1. Definitions and quantification of AF burden;
2. Associations between AF burden and clinical outcomes, including ischemic stroke, heart failure, cognitive decline, dementia, and quality of life;
3. Anticoagulation strategies in subclinical or device-detected AF;
4. Catheter ablation and AF burden reduction; and
5. Emerging technologies and future directions in AF burden assessment and individualized anticoagulation strategies.

Duplicate publications, conference abstracts without full peer-reviewed manuscripts, and studies with insufficient methodological detail were excluded.

Given the rapidly evolving nature of AF burden research, a narrative approach was considered more appropriate for integrating emerging evidence, conceptual developments, and evolving clinical perspectives. Formal risk-of-bias assessment and quantitative meta-analysis were therefore not performed.

The aim of this review was to provide a clinically oriented synthesis of current evidence and evolving conceptual frameworks related to AF burden, integrated thromboembolic and bleeding risk assessment, and individualized anticoagulation decision-making.

## 3 Definition and measurement of atrial fibrillation burden

### 3.1 Standardized definitions of AF burden–related terminology

Terminology related to atrial fibrillation (AF) burden and device-detected atrial arrhythmias remains heterogeneous across studies and clinical guidelines. Terms such as atrial high-rate episodes (AHREs), subclinical atrial fibrillation (SCAF), device-detected AF, and clinical AF are frequently used inconsistently, which complicates cross-study comparisons and interpretation of clinical evidence (1, 30). In the present review, terminology was standardized primarily according to the 2025 ESC Council on Stroke/European Heart Rhythm Association consensus statement and the American Heart Association scientific statement on subclinical and device-detected AF (1, 30). Standardized definitions adopted in this review are summarized in Supplementary Table S1.

### 3.2 Evolution of the definition of AF burden

AF burden is generally defined as the proportion of monitored time spent in AF during a defined monitoring period, preferably reported together with the duration of the longest uninterrupted AF episode when clinically relevant (30). Unlike traditional classifications based on episode duration and clinical presentation—such as paroxysmal AF, persistent AF, and permanent AF—AF burden conceptualizes AF as a dynamic and continuous process, emphasizing temporal AF exposure rather than a binary diagnostic classification.

The concept of AF burden largely originated from studies using implantable cardiac rhythm monitoring devices. The TRENDS study was among the first prospective investigations to apply the metric of “daily AF burden,” defined as the cumulative duration of AF or atrial tachyarrhythmia within a 24-hour period (11). Subsequently, the ASSERT study adopted a similar approach by quantifying the duration of device-detected AHREs (9). These studies established AF burden as a measurable quantitative construct; however, substantial heterogeneity in burden definitions and reporting methods remains across studies (30).

### 3.3 Operational metrics of AF burden

In the existing literature, AF burden has been quantified using several operational approaches, including the duration of individual AF episodes, cumulative AF duration over a defined time interval, and the proportion of monitored time spent in AF. The TRENDS study used cumulative daily AF burden and identified ≥5.5 h/day as a stratification category associated with higher thromboembolic risk (11). In contrast, ASSERT and subsequent analyses mainly evaluated AHRE duration categories, commonly including episodes lasting ≥6 min or ≥24 h (10). The KP-RHYTHM study further analyzed AF burden as a continuous variable and demonstrated an independent association between increasing AF burden and ischemic stroke risk after adjustment for CHA_2_DS_2_-VASc score (12).

Importantly, these burden categories and duration cutoffs were primarily developed for observational stratification and risk analysis rather than as universal therapeutic thresholds for anticoagulation decision-making. Variability in operational definitions across studies substantially limits direct comparisons of AF burden values and associated risk estimates (29).

### 3.4 Impact of monitoring modalities on AF burden measurement

Assessment of AF burden is highly dependent on the rhythm monitoring modality used. Implantable pacemakers, implantable cardioverter-defibrillators, and insertable cardiac monitors enable long-term continuous rhythm monitoring and are generally regarded as the reference standard for AF burden quantification (4). By contrast, short-term Holter monitoring and intermittent electrocardiographic recordings may substantially underestimate true AF burden because of limited monitoring duration (31).

In recent years, wearable devices have been increasingly incorporated into AF screening and rhythm surveillance strategies (7, 8). However, important variability remains regarding monitoring continuity, signal quality, algorithm performance, and AF adjudication methods. Current evidence supporting wearable-derived AF burden measurements is still largely based on validation studies, whereas prospective outcome-based evidence remains limited (7, 8). As wearable technologies become increasingly integrated into population-level AF screening and longitudinal rhythm surveillance, future AF burden assessment will likely rely more heavily on wearable-derived rhythm data obtained outside traditional clinical settings.

### 3.5 Heterogeneity in AF burden measurement and methodological implications

Although AF burden has been widely investigated in studies evaluating thromboembolic risk, substantial heterogeneity in burden definitions and measurement methods continues to limit comparability across studies (35). Differences in monitoring duration, burden calculation methods, episode adjudication, and analytical thresholds complicate interpretation of AF burden across different clinical settings (30). Furthermore, most existing studies rely on AF burden measured during a baseline monitoring period and do not fully account for temporal variability or longitudinal changes in AF burden over time (14). Emerging evidence suggests that AF burden is dynamic rather than static, with considerable intraindividual variation in episode frequency, duration, and temporal clustering (14, 35). Accordingly, AF burden may be better viewed as a continuous and evolving marker of atrial arrhythmia exposure rather than as a fixed categorical variable (35).

In summary, AF burden provides an important quantitative framework for characterizing AF exposure and temporal arrhythmia dynamics. However, heterogeneity in monitoring methods, burden definitions, and analytical approaches currently limits the direct translation of AF burden metrics into anticoagulation decision-making (4, 30). Current evidence suggests that AF burden should be interpreted within an integrated and individualized clinical framework that also incorporates thromboembolic risk, bleeding risk, comorbidities, and monitoring context, rather than as an isolated determinant of treatment decisions. Future studies should therefore focus not only on standardizing AF burden assessment, but also on clarifying how AF burden may complement conventional risk stratification in guiding dynamic evaluations of net clinical benefit.

Major observational and conceptual studies evaluating AF burden and its clinical implications are summarized in Table 1.

**Table 1:** Major observational and conceptual studies evaluating atrial fibrillation burden and its clinical implications.

Study	Study population/design	Monitoring modality	Definition or focus of AF burden	Main findings	Clinical implication
van Vreeswijk et al., 2026 (5)	Patients undergoing continuous rhythm monitoring	Implantable continuous monitoring	Irregular atrial arrhythmias lasting <30 s	Very short atrial arrhythmias were associated with future AF development	Suggested potential prognostic significance of ultra-low AF burden and very short arrhythmias
ESC/EHRA Consensus (Doehner et al., 2025) (30)	ESC/EHRA expert consensus statement	Multiple monitoring modalities	Standardization of AF burden definition and reporting	Proposed defining AF burden primarily as the proportion of monitored time spent in AF and recommended reporting the longest uninterrupted AF episode	Promoted standardization and comparability of AF burden assessment across studies
Doundoulakis et al., 2025 (35)	Conceptual review and expert analysis	Multiple monitoring modalities	Moving beyond categorical AF classification	Emphasized AF burden as a continuous spectrum rather than a binary “AF/no AF” entity	Provided conceptual support for burden-based clinical frameworks
Peigh et al., 2024 (14)	Patients undergoing longitudinal AF monitoring	Implantable continuous monitoring	Dynamic changes in maximum daily AF burden	Increasing AF burden over time was associated with adverse clinical outcomes	Highlighted the importance of temporal variability and AF burden trajectories
Singer et al., 2021 (13)	Patients with continuously monitored AF and ischemic stroke events	Implantable continuous monitoring	Temporal association between AF episodes and stroke occurrence	Stroke events were not consistently temporally related to AF episodes	Suggested AF burden may reflect an underlying prothrombotic substrate rather than an immediate stroke trigger
KP-RHYTHM (Go et al., 2018) (12)	Patients with paroxysmal AF undergoing ambulatory monitoring	Continuous ambulatory ECG monitoring	Percentage of monitored time spent in AF (continuous variable)	AF burden remained independently associated with ischemic stroke risk after adjustment for CHA_2_DS_2_-VASc score	Supported AF burden as an independent quantitative risk marker
Van Gelder et al., 2017 (10)	Secondary analysis of the ASSERT cohort	Implantable continuous monitoring	Different AHRE duration thresholds (e.g., ≥6 min, ≥24 h)	Longer AF episode duration was associated with progressively higher stroke risk	Suggested a duration-dependent relationship between AF burden and thromboembolic risk
ASSERT (Healey et al., 2012) (9)	Patients with implanted pacemakers or defibrillators without prior clinical AF	Implantable continuous monitoring	Device-detected atrial high-rate episodes (AHREs) ≥ 6 min	Subclinical AF was associated with an increased risk of ischemic stroke or systemic embolism	Established the prognostic significance of device-detected subclinical AF
TRENDS (Glotzer et al., 2009) (11)	Patients with implantable cardiac devices and stroke risk factors	Implantable continuous monitoring	Daily cumulative AF burden; high burden defined as ≥5.5 h/day	Higher AF burden was associated with increased thromboembolic risk	Introduced cumulative daily AF burden as a clinically relevant quantitative metric

## 4 Clinical implications of AF burden

### 4.1 AF burden and ischaemic stroke

Ischaemic stroke remains the most clinically important adverse outcome associated with atrial fibrillation (AF). Studies using continuous rhythm monitoring have consistently shown that greater AF burden is associated with a higher risk of stroke and systemic embolism (29). In the TRENDS study, patients with device-detected AF burden ≥5.5 h/day had a higher thromboembolic risk than those with lower AF burden (11). Subsequent analyses from ASSERT further demonstrated that longer durations of device-detected atrial high-rate episodes (AHREs) were associated with progressively higher stroke risk (10). Similarly, the KP-RHYTHM study showed that AF burden remained independently associated with stroke risk after adjustment for the CHA_2_DS_2_-VASc score (12).

Importantly, these studies were observational and primarily demonstrated risk associations rather than direct treatment effects (31). In many patients with low AF burden, the absolute stroke risk remained relatively low (15). In addition, temporal analyses showed that stroke events were often not temporally linked to AF episodes themselves, suggesting that AF burden may partly reflect an underlying atrial cardiomyopathic or prothrombotic substrate rather than serving solely as an immediate trigger for thromboembolism (13).

Current evidence therefore supports AF burden as a complementary marker within integrated thromboembolic risk assessment rather than as an isolated determinant of anticoagulation decisions. AF burden should be interpreted together with established clinical risk factors, particularly the CHA_2_DS_2_-VASc score, patient comorbidities, and overall bleeding risk.

### 4.2 AF burden and heart failure

AF and heart failure frequently coexist and share a complex bidirectional relationship (21). Available evidence suggests that higher AF burden may be associated with worsening cardiac function and increased heart failure–related hospitalization (4, 20). Continuous rhythm monitoring studies have shown that patients with greater AF burden are more likely to experience adverse structural and functional cardiac remodeling (4, 20).

Several mechanisms may contribute to this association, including loss of atrial mechanical function, irregular ventricular response, impaired rate control, and progression of atrial and ventricular remodeling (21). However, current evidence is derived mainly from observational studies and *post hoc* analyses. Whether AF burden independently contributes to heart failure progression or primarily reflects more advanced cardiovascular disease remains uncertain.

At present, no adequately powered randomized trial has demonstrated that reducing AF burden alone directly lowers heart failure events (4, 20). Accordingly, AF burden should currently be regarded as a dynamic marker associated with heart failure risk rather than a standalone therapeutic target for heart failure prevention.

### 4.3 AF burden, cognitive decline, and dementia

Increasing evidence suggests that AF may contribute to cognitive decline and dementia, even in the absence of clinically recognized stroke (40). This has generated growing interest in whether cumulative AF exposure may influence long-term neurocognitive outcomes.

Continuous monitoring studies have explored the relationship between AF burden and cognitive performance. Bonnesen et al. evaluated elderly individuals undergoing implantable loop recorder monitoring and longitudinal cognitive assessment using the Montreal Cognitive Assessment (MoCA) (38). After adjustment for vascular risk factors, AF burden was not independently associated with significant cognitive decline during follow-up. However, the study demonstrated the feasibility of combining continuous rhythm monitoring with serial neurocognitive evaluation.

In contrast, Tang et al. reported that patients with higher AF burden during prolonged ECG monitoring had significantly lower MoCA scores, even after adjustment for cardiovascular comorbidities and echocardiographic parameters (39). These findings suggest that greater AF exposure may contribute to impaired cognitive performance independently of conventional stroke risk factors.

Several mechanisms have been proposed to explain the association between AF burden and neurocognitive impairment, including silent cerebral infarction, cerebral hypoperfusion, endothelial dysfunction, systemic inflammation, and impaired cerebral autoregulation (40). These mechanisms support the hypothesis that cumulative AF exposure may contribute to progressive cerebral injury over time.

The BRAIN-AF randomized trial evaluated whether anticoagulation could reduce cognitive decline in patients with AF and relatively low thromboembolic risk (41). Rivaroxaban did not reduce the composite outcome of cognitive decline, stroke, or transient ischaemic attack compared with placebo. Although the study was terminated early because of futility, it highlighted cognition as an important clinical outcome beyond traditional stroke prevention.

Despite these observations, major uncertainties remain. Most available evidence is observational, AF burden definitions vary substantially across studies, and the temporal relationship between AF burden progression and cognitive decline is incompletely understood. Whether reduction of AF burden through rhythm-control strategies improves long-term cognitive outcomes has not yet been established in randomized trials. Overall, current evidence suggests that higher AF burden may be associated with cognitive decline and dementia, but causality remains uncertain. AF burden should therefore be interpreted as part of a broader individualized clinical framework rather than as an independent neurocognitive risk threshold.

### 4.4 AF burden and quality of life

Symptom burden and quality of life are major treatment goals in AF management. The CABANA trial demonstrated that catheter ablation improved AF-related quality of life more effectively than medical therapy in symptomatic AF patients (23). Using the Atrial Fibrillation Effect on Quality-of-Life (AFEQT) questionnaire (26), patients undergoing ablation reported significantly better symptom control and functional status during follow-up (23).

Subsequent studies have further explored the relationship between AF burden reduction and quality-of-life improvement. In the CIRCA-DOSE trial, continuous rhythm monitoring demonstrated that larger reductions in AF burden after catheter ablation were associated with greater improvements in AFEQT scores (24). These findings support the concept that AF burden may correlate more closely with patient-reported outcomes than conventional binary AF recurrence measures.

Similar observations have been reported in persistent AF populations. Yeo et al. found that lower post-ablation AF burden was associated with better physical and social functioning as assessed by SF-36 questionnaires (25). Together, these studies suggest that AF burden may provide clinically meaningful information regarding symptom severity and quality-of-life outcomes.

However, several limitations should be acknowledged. Definitions of AF burden and monitoring strategies vary substantially across studies, and randomized trials specifically using AF burden as a primary therapeutic target remain limited. In addition, quality-of-life improvement is influenced by multiple clinical factors beyond AF burden alone, including symptom perception, comorbidities, heart failure status, and psychological factors.

Overall, available evidence supports an association between AF burden and several clinically relevant outcomes, particularly ischaemic stroke. Associations with heart failure, cognitive decline, dementia, and quality of life have also been reported, although most evidence outside stroke prevention remains observational. Importantly, these findings support the integration of AF burden into individualized and context-dependent clinical assessment rather than its use as an isolated determinant of treatment decisions.

## 5 Clinical trials and interpretation of anticoagulation benefit in AF

### 5.1 From risk association to anticoagulation decision-making

Observational studies such as ASSERT, TRENDS, and KP-RHYTHM consistently demonstrated that increasing AF burden is associated with higher thromboembolic risk (9, 11, 12). However, these studies primarily evaluated risk association rather than the balance between treatment benefit and harm (29). Importantly, an association between AF burden and stroke risk does not necessarily imply that oral anticoagulation provides equivalent clinical benefit across all levels of AF exposure.

In patients with device-detected AF, subclinical atrial fibrillation (SCAF), or atrial high-rate episodes (AHREs), the absolute risk of ischaemic stroke is often relatively low, particularly in individuals with limited AF exposure. In contrast, the bleeding risk associated with oral anticoagulation does not appear to decrease proportionally with lower levels of monitored AF burden. This imbalance suggests that the net clinical benefit of anticoagulation may vary substantially across different clinical contexts rather than being uniformly distributed across the spectrum of AF exposure (18, 33).

Current evidence therefore supports a more integrated interpretation of anticoagulation benefit, in which AF burden should be considered together with conventional thromboembolic risk assessment, bleeding risk, comorbidities, age, frailty, and patient-specific clinical characteristics. Within this framework, AF burden may function as a complementary marker of risk rather than an isolated determinant of anticoagulation therapy.

### 5.2 LOOP: anticoagulation in populations with limited AF exposure

The LOOP study was a prospective randomized trial that enrolled elderly individuals without previously diagnosed AF but with elevated stroke risk and evaluated a screening strategy using insertable cardiac monitors (15). Oral anticoagulation was initiated after detection of device-detected AF. Most AF episodes identified during follow-up were brief, and the overall monitored AF exposure within the study population was relatively limited (15).

Compared with usual care, continuous monitoring substantially increased AF detection and anticoagulation use but did not significantly reduce the incidence of stroke or systemic embolism (15). These findings suggest that in populations characterized predominantly by limited AF exposure and relatively low absolute stroke event rates, anticoagulation initiated solely on the basis of AF detection may provide limited overall clinical benefit.

Importantly, LOOP was not designed to evaluate anticoagulation efficacy according to predefined AF burden strata. Therefore, the results should not be interpreted as evidence that anticoagulation is ineffective in all patients with limited AF exposure. Rather, the study highlights the complexity of translating device-detected AF into clinically meaningful anticoagulation decisions within an individualized and integrated risk framework.

### 5.3 ARTESiA: modest Net clinical benefit in device-detected SCAF

The ARTESiA trial was a randomized double-blind study comparing apixaban with aspirin in patients with device-detected SCAF and elevated stroke risk (17). Most enrolled patients had relatively limited monitored AF exposure and did not meet conventional criteria for clinically diagnosed AF.

Apixaban reduced the incidence of ischaemic stroke and systemic embolism but significantly increased major bleeding events (17). As a result, the overall net clinical benefit at the population level appeared modest, particularly in individuals with lower baseline thromboembolic risk.

Secondary analyses from ARTESiA further demonstrated that baseline AF burden, including episode frequency and longest episode duration, was not independently associated with stroke or systemic embolism risk and did not significantly modify the relative efficacy of apixaban (37). These findings suggest that AF burden alone may not adequately identify patients who derive greater benefit from anticoagulation therapy.

Collectively, ARTESiA supports a more integrated and context-dependent approach to anticoagulation decision-making, in which AF burden complements rather than replaces established thromboembolic risk assessment.

### 5.4 NOAH-AFNET 6: limited anticoagulation benefit in AHRE populations

The NOAH-AFNET 6 trial evaluated edoxaban vs. placebo in patients with device-detected AHREs (6) without clinically documented AF (16). Similar to ARTESiA, the study population was characterized predominantly by short-duration atrial arrhythmias and relatively limited monitored AF exposure.

The trial did not demonstrate a significant reduction in the composite outcome of cardiovascular death, stroke, or systemic embolism with anticoagulation, whereas major bleeding and adverse safety events increased significantly (16). The trial was terminated early because of concerns regarding safety and futility.

Supplementary analyses from NOAH-AFNET 6 showed no significant interaction between baseline AHRE burden and the treatment effect of anticoagulation (16). Together with findings from ARTESiA, these data suggest that currently available randomized evidence does not support the use of baseline AF burden alone to determine anticoagulation benefit in patients with device-detected atrial arrhythmias.

Importantly, differences in study design, inclusion criteria, and comparator strategies between ARTESiA and NOAH-AFNET 6 limit direct comparison of the two trials. Nevertheless, both studies consistently emphasize that anticoagulation benefit in device-detected AF populations is influenced by the broader clinical context rather than isolated rhythm-related parameters.

### 5.5 An integrated framework for net clinical benefit

When observational studies and randomized trials are considered collectively, the relationship between AF burden and anticoagulation benefit appears dynamic rather than binary. Current evidence suggests that the net clinical benefit of oral anticoagulation varies according to the interaction among AF burden, baseline thromboembolic risk, bleeding risk, and broader patient-specific clinical characteristics.

**Figure 1:**
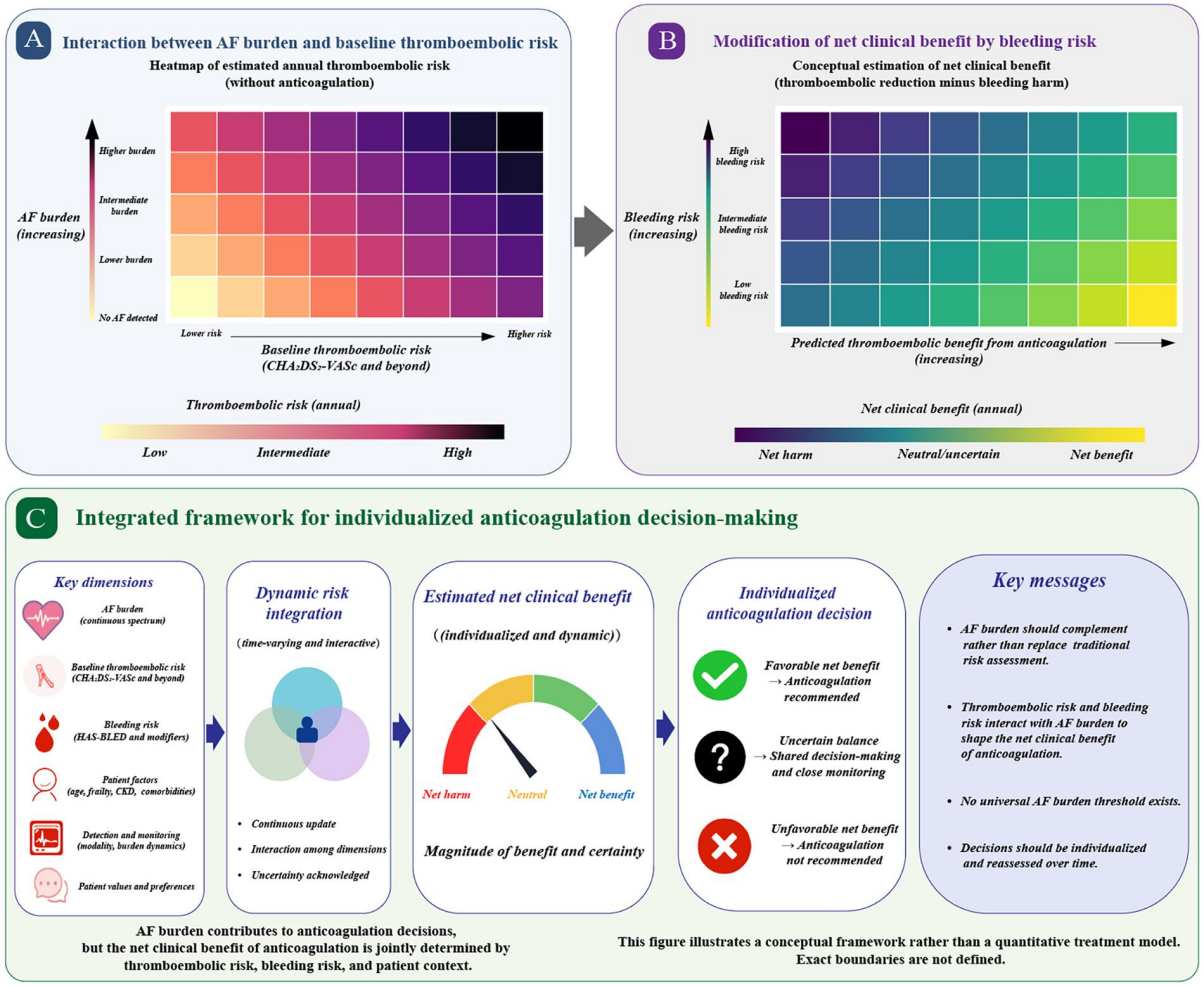
AF burden and anticoagulation: a context-dependent framework for net clinical benefit. This figure illustrates a clinically oriented conceptual framework integrating atrial fibrillation (AF) burden, thromboembolic risk, and bleeding risk in the evaluation of the net clinical benefit of oral anticoagulation (OAC). AF burden is defined as the proportion of monitored time spent in AF during a defined monitoring period, preferably reported together with the longest uninterrupted AF episode when relevant. AF burden should be interpreted together with conventional clinical risk assessment rather than as an isolated determinant of anticoagulation therapy. Panel A illustrates the interaction between AF burden and baseline thromboembolic risk, represented by the CHA_2_DS_2_-VASc score. Increasing AF burden in combination with higher baseline thromboembolic risk is conceptually associated with progressively greater estimated thromboembolic risk in the absence of anticoagulation therapy. Panel B illustrates modification of the estimated net clinical benefit of anticoagulation by bleeding risk, represented by the HAS-BLED score. Bleeding risk associated with anticoagulation may remain clinically relevant across different levels of AF burden and may vary substantially according to comorbidities, frailty, concomitant therapies, and other patient-specific clinical characteristics. Panel C summarizes the multidimensional integration of AF burden, thromboembolic risk, bleeding risk, and patient context within individualized anticoagulation decision-making. Within this framework, the estimated net clinical benefit of anticoagulation is dynamic and context-dependent rather than binary or determined by a universal AF burden threshold. Patients with limited monitored AF burden and low thromboembolic risk may derive limited overall benefit from anticoagulation, whereas individuals with greater monitored AF burden, elevated thromboembolic risk, and acceptable bleeding risk may be more likely to experience a favorable balance between thromboembolic reduction and bleeding risk. This figure is intended as a conceptual and explanatory framework integrating findings from observational studies and randomized clinical trials. It is not intended to define specific thresholds for initiation, modification, or discontinuation of anticoagulation therapy. Clinical decision-making should continue to incorporate integrated assessment of thromboembolic risk, bleeding risk, comorbidities, rhythm-monitoring profiles, patient characteristics, and patient preferences. No universal AF burden threshold exists, and individualized assessment remains essential.

Figure 1 summarizes this integrated conceptual framework, illustrating how AF burden, CHA_2_DS_2_-VASc score, and bleeding risk may interact dynamically to influence the net clinical benefit of anticoagulation. Within this model, patients with limited AF exposure and low thromboembolic risk may derive limited absolute stroke reduction from anticoagulation, whereas bleeding risk may remain clinically relevant. In contrast, individuals with greater AF exposure in combination with elevated thromboembolic risk and acceptable bleeding risk may be more likely to experience a favorable balance between benefit and harm.

Importantly, this framework should not be interpreted as evidence for a universal AF burden threshold for initiating or discontinuing anticoagulation therapy. Rather, it is intended as a conceptual model illustrating that the relationship between AF burden and net clinical benefit is likely continuous, individualized, and context-dependent.

An additional challenge in interpreting net clinical benefit is that thromboembolic and bleeding events are not clinically equivalent outcomes. Ischaemic stroke, systemic embolism, intracranial haemorrhage, and non-fatal major bleeding differ substantially in terms of mortality, disability, long-term functional consequences, and patient-perceived impact. Consequently, net clinical benefit should not be viewed as a simple arithmetic balance between event counts alone.

Furthermore, the relative importance assigned to stroke prevention vs. bleeding risk may vary considerably across patients, clinicians, and healthcare systems. Frailty, comorbidities, life expectancy, functional status, and quality-of-life considerations may all influence individualized treatment preferences. These considerations also highlight the importance of shared decision-making in anticoagulation management for patients with device-detected AF or SCAF.

These complexities further support the use of an integrated and individualized decision-making framework rather than reliance on isolated AF burden values or simplified threshold-based models.

At present, no randomized controlled trial has prospectively stratified anticoagulation therapy according to continuously quantified AF burden with the explicit goal of testing burden-dependent treatment benefit. Accordingly, the current framework remains hypothesis-generating and primarily serves as a clinically oriented interpretation of existing evidence rather than a basis for defining actionable treatment cutoffs. This issue may become increasingly important with the expanding use of wearable and consumer-based rhythm-monitoring technologies, which are expected to substantially increase detection of intermittently monitored atrial arrhythmias in the future.

### 5.6 AF burden, catheter ablation, and anticoagulation strategy

Catheter ablation consistently reduces AF recurrence and overall AF burden; however, its implications for long-term anticoagulation strategies remain uncertain (22). Periprocedural anticoagulation strategies during AF ablation appear to be safe under uninterrupted direct oral anticoagulant regimens, as demonstrated in real-world registry data (42). In the CABANA trial, catheter ablation improved quality of life and symptom burden compared with medical therapy, although stroke outcomes and anticoagulation modification were not primary study endpoints (23).

Similarly, the CIRCA-DOSE trial and subsequent prospective studies demonstrated that reductions in AF burden after ablation were associated with improvements in symptoms and quality of life (24, 25). Continuous rhythm monitoring studies further showed that clinically meaningful improvements may occur even in patients classified as having conventionally defined AF recurrence according to binary rhythm definitions (36). These findings support the concept that AF-related clinical outcomes may be better understood along a continuum of AF burden rather than solely through dichotomous rhythm classifications.

However, no randomized trial has systematically evaluated anticoagulation discontinuation or adjustment strategies based on post-ablation AF burden. Consequently, despite substantial reductions in AF burden after successful ablation, current evidence does not support withdrawal of oral anticoagulation solely on the basis of reduced AF burden.

Current guidelines and available evidence therefore continue to support anticoagulation decisions primarily based on integrated thromboembolic risk assessment, while AF burden may serve as a complementary marker within individualized clinical decision-making. Real-world registry data further suggest variability in post-ablation anticoagulation strategies and outcomes, highlighting ongoing uncertainty regarding optimal anticoagulation management after ablation (42).

### 5.7 Summary

Current randomized evidence indicates that, in patients with device-detected atrial arrhythmias or SCAF, oral anticoagulation provides modest reduction in ischaemic stroke risk at the expense of increased bleeding risk, resulting in a limited or variable net clinical benefit at the population level.

Findings from ARTESiA and NOAH-AFNET 6 consistently suggest that anticoagulation benefit is influenced predominantly by overall thromboembolic risk and clinical context rather than by AF burden alone. Importantly, neither trial was specifically designed to evaluate anticoagulation strategies based on continuously quantified AF burden.

Taken together, currently available evidence supports continued use of established stroke risk assessment as the primary basis for anticoagulation therapy. AF burden may provide additional complementary information within a dynamic and individualized framework integrating thromboembolic risk, bleeding risk, patient characteristics, and rhythm-monitoring profiles. The role of AF burden as an independent determinant of anticoagulation benefit remains unproven and requires further validation in prospective studies specifically designed to incorporate longitudinal AF burden assessment within integrated risk-based treatment frameworks.

Key randomized and prospective studies evaluating anticoagulation strategies in subclinical or low-burden AF are summarized in Table 2.

**Table 2:** Major randomized, secondary, and prospective studies evaluating anticoagulation strategies in subclinical or Low-burden atrial fibrillation.

Trial	Population	AF/AHRE Definition	AF Burden Characteristics	Intervention	Comparator	Main Efficacy Outcome	Major Bleeding Outcome	Interaction by AF burden	Main Interpretation
Winstén et al., 2025 (Net Clinical Benefit Analyses) (18, 33)	Patients with device-detected subclinical AF from observational and registry-based cohorts	Device-detected AF/AHRE	Predominantly low-burden AF populations	Oral anticoagulation exposure analyses	No anticoagulation	Suggested modest reduction in thromboembolic risk in selected higher-risk populations	Bleeding risk offset part of the benefit	No consistent interaction between AF burden and net clinical benefit; net benefit appeared more dependent on baseline stroke risk than AF burden alone	Supports individualized anticoagulation strategies integrating stroke and bleeding risk
McIntyre et al., 2024 (ARTESiA Secondary Analysis) (37)	Secondary analyses from the ARTESiA cohort	Baseline device-detected subclinical AF burden	Quantitative burden-based subgroup analyses	Apixaban	Aspirin	No clear relationship between baseline AF burden and stroke reduction efficacy	Bleeding increase remained present	No significant interaction between baseline AF burden and apixaban efficacy; baseline AF burden was not independently associated with stroke risk	Findings challenge the concept that AF burden alone determines anticoagulation benefit
Healey et al., 2024 (ARTESiA) (17)	Patients with device-detected subclinical AF and elevated stroke risk	Subclinical AF episodes ≥6 min but <24 h	Low-burden subclinical AF identified by implanted devices	Apixaban	Aspirin	Reduced stroke or systemic embolism	Increased major bleeding	Primary publication did not establish AF burden-dependent treatment effects; benefit-risk balance remained modest at the population level	Anticoagulation reduced thromboembolic events at the expense of increased bleeding
Kirchhof et al., 2023 (NOAH-AFNET 6 Supplementary Analysis) (16)	Subgroups derived from the NOAH-AFNET 6 population	Baseline AHRE burden stratification	Burden-stratified analyses across low- and higher-burden AHRE groups	Edoxaban	Placebo/usual care	No differential efficacy according to baseline AF burden	Bleeding risk remained increased across burden strata	No significant interaction between baseline AF burden and treatment effect; anticoagulation effects were generally consistent irrespective of baseline AF burden	Current evidence does not support AF burden alone as a determinant of anticoagulation benefit
Kirchhof et al., 2023 (NOAH-AFNET 6) (16)	Patients with atrial high-rate episodes (AHRE) without ECG-documented AF	AHRE ≥6 min detected by implanted devices	Mostly short-duration, low-burden AHRE/subclinical AF	Edoxaban	Placebo/usual care	No significant reduction in cardiovascular death, stroke, or systemic embolism	Increased major bleeding and safety events	Primary publication did not formally report burden-treatment interaction analysis; overall efficacy of anticoagulation was limited despite increased bleeding risk	Routine anticoagulation for AHRE without clinical AF demonstrated limited net clinical benefit
Svendsen et al., 2021 (LOOP) (15)	Elderly patients with stroke risk factors undergoing implantable loop recorder screening; no prior clinical AF	Device-detected AF episodes ≥6 min	Predominantly low-burden, short-duration AF detected through continuous monitoring	Oral anticoagulation initiated after AF detection	Usual care	No significant reduction in stroke or systemic embolism	No major excess in bleeding reported	Interaction analysis not formally performed; stroke risk appeared relatively low in short-duration AF episodes	Screening increased AF detection and anticoagulation use, but did not significantly reduce stroke incidence
Van Gelder et al., 2017 (ASSERT Duration Analysis) (10)	ASSERT population with duration-based AF analysis	Device-detected AF stratified by episode duration	Longer AF duration associated with progressively higher stroke risk	Observational study	N/A	Longer AF duration associated with increased stroke/systemic embolism risk	N/A	Stroke risk increased substantially with longer AF episodes	Suggested that AF burden/duration may provide incremental prognostic value beyond binary AF classification
Healey et al., 2012 (ASSERT) (9)	Patients with implanted pacemakers/defibrillators and no prior AF	Subclinical AF episodes ≥6 min	Predominantly short-duration subclinical AF	Observational study	N/A	Subclinical AF associated with increased stroke risk	N/A	Presence of device-detected AF associated with increased thromboembolic risk	First landmark study linking device-detected subclinical AF with stroke risk
Glotzer et al., 2009 (TRENDS) (11)	Patients with cardiac implantable electronic devices and stroke risk factors	Device-detected atrial tachyarrhythmia/AF	Daily AF burden ≥5.5 h associated with increased thromboembolic risk	Observational study	N/A	Higher AF burden associated with increased thromboembolic events	N/A	Suggested a dose-response relationship between AF burden and thromboembolic risk	Provided early evidence supporting AF burden as a continuous risk marker

AF, atrial fibrillation; AHRE, atrial high-rate episode; ILR, implantable loop recorder; OAC, oral anticoagulation; N/A, not applicable.

1. **AHRE** refers to device-detected atrial high-rate episodes that may not always represent electrocardiographically confirmed atrial fibrillation.

2. **AF burden** was generally defined as cumulative AF duration or proportion of monitored time spent in AF during a specified monitoring period, in accordance with recent ESC/EHRA consensus recommendations.

3. **Net clinical benefit** should be interpreted as the balance between thromboembolic risk reduction and bleeding liability, which may vary according to baseline clinical risk profiles.

## 6 Future perspectives and unresolved questions

### 6.1 Lack of standardization in AF burden definitions and measurement

Although increasing evidence supports an association between atrial fibrillation (AF) burden and adverse clinical outcomes, standardized definitions and measurement approaches for AF burden remain lacking across studies and monitoring platforms (29). Considerable heterogeneity exists in AF burden definitions, monitoring duration, reporting metrics, and rhythm-monitoring modalities, limiting direct comparisons between studies and reducing the generalizability of existing findings (35).

For example, the ASSERT study defined atrial high-rate episodes (AHREs) using episode durations ≥6 min, whereas the TRENDS study evaluated cumulative AF burden and identified higher thromboembolic risk in patients with AF burden ≥5.5 h per day (9, 11). Importantly, these values were proposed for within-study stratification and should not be interpreted as universal thresholds for anticoagulation decision-making.

In addition, differences between implantable devices, wearable monitoring technologies, intermittent electrocardiographic monitoring, and continuous rhythm-monitoring systems further complicate AF burden quantification and interpretation (7, 8). Future international efforts should prioritize harmonization of AF burden definitions, reporting standards, and monitoring methodologies to improve comparability and clinical interpretability.

### 6.2 Temporal dynamics of AF burden remain insufficiently characterized

AF burden is inherently dynamic and may vary substantially over time within the same individual (1). Single-time-point or short-duration monitoring may therefore fail to reflect long-term AF exposure. In contrast, thromboembolic risk is likely driven by cumulative and time-varying processes rather than isolated rhythm episodes (14).

Most existing studies rely on baseline AF burden or fixed monitoring windows and do not adequately account for longitudinal changes in AF burden trajectories (14). Consequently, the relationship between evolving AF burden patterns and clinical outcomes remains incompletely understood.

Future research should incorporate longitudinal and repeated rhythm monitoring to better characterize AF burden trajectories and their associations with thromboembolic risk, bleeding outcomes, cognitive function, heart failure progression, and quality of life. These approaches may support more individualized and adaptive risk assessment frameworks.

### 6.3 Lack of prospective evidence for integrated AF burden–based anticoagulation strategies

Randomized trials such as ARTESiA and NOAH-AFNET 6 have provided important evidence regarding anticoagulation in device-detected subclinical AF and atrial high-rate episode (AHRE) populations (16, 17). However, these studies were not designed to evaluate anticoagulation strategies based on continuously quantified AF burden within an integrated risk framework.

No randomized trial has prospectively stratified anticoagulation therapy according to AF burden profiles combined with thromboembolic and bleeding risk assessment. Therefore, current evidence remains insufficient to determine whether specific AF burden patterns are consistently associated with favorable net clinical benefit from anticoagulation therapy.

Rather than supporting universal thresholds, existing data suggest that the clinical relevance of AF burden is context-dependent and influenced by thromboembolic risk, bleeding risk, comorbidities, frailty, and patient-specific characteristics. Future studies should adopt integrated, multidimensional designs incorporating longitudinal AF burden assessment and dynamic treatment strategies.

### 6.4 Challenges in integrating AF burden into anticoagulation decision-making

#### 6.4.1 AF burden should complement rather than replace traditional risk assessment

The clinical significance of AF burden varies substantially according to baseline thromboembolic risk. AF-related stroke risk is strongly influenced by age, prior stroke, vascular disease, and comorbidities rather than rhythm characteristics alone (32).

Some patients with limited AF burden may still have high thromboembolic risk, while others with greater AF burden may have low absolute risk due to favorable clinical profiles (32). These observations support the concept that AF burden should complement rather than replace established risk scores such as CHA_2_DS_2_-VASc in anticoagulation decision-making.

#### 6.4.2 Dynamic nature of bleeding risk and net clinical benefit

Bleeding risk associated with oral anticoagulation is dynamic and influenced by age, renal dysfunction, frailty, prior bleeding, intracranial hemorrhage, and concomitant therapies. Accordingly, net clinical benefit should be understood as a continuously evolving balance between thromboembolic prevention and bleeding risk rather than a fixed treatment effect. This dynamic interaction is particularly relevant in patients with device-detected or limited AF burden, in whom both thromboembolic risk and bleeding risk may shift over time.

#### 6.4.3 Challenges in weighting thromboembolic prevention against bleeding risk

An additional challenge relates to the weighting of thromboembolic prevention vs. bleeding harm in net clinical benefit frameworks. Clinical outcomes differ substantially in severity, with intracranial hemorrhage and disabling stroke carrying disproportionately high clinical impact.

Moreover, patient preferences regarding acceptable trade-offs between stroke prevention and bleeding risk are highly heterogeneous. Future studies should incorporate patient-centered and severity-weighted approaches to better reflect real-world decision-making.

#### 6.4.4 Future directions for individualized anticoagulation frameworks

The increasing use of wearable devices and continuous rhythm-monitoring technologies is expanding detection of device-detected AF and low-burden atrial arrhythmias (7, 8). However, the clinical significance of very short-duration AF episodes remains uncertain (5).

Future research should integrate longitudinal AF burden trajectories, multimodal monitoring, biomarkers, and clinical risk profiles into individualized and context-dependent anticoagulation frameworks. Large prospective registries and real-world cohort studies may complement randomized trials by evaluating feasibility and safety in routine practice.

Artificial intelligence–based analytics and digital health technologies may further enable dynamic risk prediction and individualized assessment of net clinical benefit.

### 6.5 Emerging technologies and digital health applications

Wearable devices, remote monitoring systems, and artificial intelligence–based tools are rapidly improving AF burden detection and characterization (7, 8). These technologies may enable near–real-time rhythm assessment, improve accessibility, and reduce monitoring burden. Future data-driven models integrating AF burden, thromboembolic risk, bleeding risk, and biomarkers may facilitate more precise and individualized stroke prevention strategies. However, challenges remain regarding data quality, validation, standardization, and clinical interpretation of device-detected atrial arrhythmias. Prospective validation is required before routine clinical implementation.

### 6.6 Implications for clinical practice and guideline development

Current evidence supports AF burden as a complementary conceptual framework linking temporal AF patterns with thromboembolic risk. However, AF burden is not sufficiently validated as an independent determinant for anticoagulation initiation, modification, or discontinuation. Instead, AF burden should be integrated with thromboembolic risk scores, bleeding risk assessment, comorbidities, and patient preferences within a context-dependent and individualized decision framework. Future guideline updates may progressively incorporate AF burden as an additional dimension within integrated risk assessment models, particularly in patients with device-detected or subclinical AF.

## Conclusion

AF burden provides a quantitative framework for characterizing the temporal exposure to atrial fibrillation. Observational studies based on continuous rhythm monitoring, including ASSERT, TRENDS, and KP-RHYTHM, have consistently demonstrated a positive association between increasing AF burden and the risk of ischemic stroke; however, absolute stroke risk remains generally low among individuals with limited AF burden.

Randomized controlled trials such as ARTESiA and NOAH-AFNET 6 have further evaluated the efficacy and safety of oral anticoagulation in patients with subclinical or device-detected AF. These trials suggest that oral anticoagulation may reduce ischemic stroke or systemic embolism, but this benefit is accompanied by an increased risk of major bleeding, resulting in a context-dependent and overall modest net clinical benefit that is largely influenced by baseline thromboembolic risk.Taken together, evidence from observational and randomized studies indicates that the net clinical benefit of anticoagulation is not uniform across different levels of AF burden, but is determined by a dynamic and individualized balance between thromboembolic risk reduction and bleeding risk. The concept of a “maximum net clinical benefit threshold” should therefore be interpreted only as a conceptual hypothesis to describe heterogeneity across studies, rather than as a clinically applicable cutoff.Accordingly, AF burden alone is insufficient to serve as a standalone determinant for anticoagulation decisions. Instead, AF burden should complement rather than replace established thromboembolic and bleeding risk assessment within an integrated and context-dependent decision-making framework.

Future studies should focus on standardizing AF burden definitions and measurement, incorporating longitudinal and dynamic assessments of AF burden, and evaluating anticoagulation strategies within prospective, risk-integrated study designs. These efforts may help advance stroke prevention in AF toward more precise, individualized, and context-aware management.
