# Supplementary material for: Atrial fibrillation burden and oral anticoagulation: a context-dependent framework for net clinical benefit beyond binary AF definitions
Source: Front Cardiovasc Med. 2026 Jul 2;13:1859191. doi: 10.3389/fcvm.2026.1859191 (PMC13372787; doi:10.3389/fcvm.2026.1859191)
Supplement: Supplementary file 1 [file Table1.docx]

**Supplementary Table 1. Standardized Terminology Related to AF Burden and Device-Detected Atrial Arrhythmias**

| **Term** | **Standardized definition** | **Key clinical implication** |
| --- | --- | --- |
| AF burden | The proportion of monitored time spent in atrial fibrillation during a defined monitoring period, preferably reported together with the longest uninterrupted AF episode when relevant | Recommended quantitative measure of AF exposure |
| LEAF (Longest Episode of AF) | The duration of the longest uninterrupted AF episode during a monitoring period | Supplementary descriptor frequently reported alongside AF burden |
| AHRE (Atrial high-rate episodes) | Device-detected atrial events, usually tachyarrhythmias, meeting programmed or specified atrial high-rate criteria | May represent AF, other atrial tachyarrhythmias, or artefacts unless adjudicated |
| SCAF (Subclinical atrial fibrillation) | Asymptomatic episodes of atrial fibrillation detected by implantable or wearable monitors and confirmed by intracardiac electrogram or ECG review | Requires rhythm-confirmed AF without symptoms |
| SCAT (Subclinical atrial tachyarrhythmia) | Asymptomatic episodes of AF, atrial flutter, or atrial tachycardia confirmed by intracardiac recordings | Broader concept than SCAF |
| DDAF (Device-detected atrial fibrillation) | Atrial fibrillation identified through implantable or wearable monitoring devices, with or without symptoms | Includes AF detected outside conventional clinical settings |
| Clinical AF | Atrial fibrillation documented by surface ECG in routine clinical practice | Conventional clinically diagnosed AF |
| Low-burden AF | A descriptive term generally referring to short-duration or low cumulative AF exposure; definitions vary substantially across studies and clinical settings | Clinical significance should be interpreted within the context of overall thromboembolic and bleeding risk rather than by a universal threshold alone |
| AF density | Temporal aggregation or clustering of AF episodes during a monitoring period | Distinct from cumulative AF burden |
| Burden-guided risk assessment | Integration of AF burden with thromboembolic risk, bleeding risk, comorbidities, and monitoring context | Supports individualized rather than threshold-based anticoagulation decision-making |

**Footnote**

Definitions were primarily adapted from the 2025 ESC Council on Stroke/EHRA clinical consensus statement on AF burden and the 2023 ACC/AHA/ACCP/HRS guideline for the management of atrial fibrillation ^[1,30]^, with terminology harmonized for consistency throughout this review.
